# Biomarkers in Melanoma: Updates in Prognosis and Management

**DOI:** 10.3390/cancers18121992

**Published:** 2026-06-18

**Authors:** Brett Crosby, Martin Guerra, Alyssa Crosby, Benjamin Linza, Kristel Lourdault, Richard Essner

**Affiliations:** Saint John’s Cancer Institute, Providence Health & Services, Division of Surgical Oncology, Borstein Family Melanoma Program, Laboratory of Cutaneous Oncology, Santa Monica, CA 90404, USA; brett.crosby@providence.org (B.C.); martin.guerra1@providence.org (M.G.); alyssa.crosby@providence.org (A.C.); benjamin.linza@providence.org (B.L.); kristel.lourdault@providence.org (K.L.)

**Keywords:** Melanoma, Biomarkers, gene expression signatures, miRNA, Nomogram, LDH, S100B

## Abstract

Melanoma incidence rates have also been steadily increasing, emphasizing the need for improved prognostic and diagnostic tools with the goal of enhancing patients’ outcomes. Biomarkers in melanoma have emerged as an important component of melanoma management, offering insight into disease progression, tumor biology, and the potential for judging treatment responses. Traditionally, blood and immunohistochemical markers such as lactate dehydrogenase (LDH), S100 calcium-binding protein (S100B), human melanoma black-45 (HMB-45), and SRY-box transcription factor 10 (SOX10) have been widely used in melanoma diagnosis, staging, and monitoring. However, their clinical use has been limited because of their low specificity, especially in patients with early-stage disease. This has led to the development of molecular and genetic biomarkers, including BRAF, NRAS, and KIT mutations, which improved patients’ risk stratification and enabled targeted therapies, and gene expression signature assays such as DecisionDx (Castle Biosciences) and SkylineDx (Merlin) that are already used in clinics to help with surgical decisions and to assess patients’ prognosis. Other circulating biomarkers, including microRNAs, circulating tumor DNA and circulating tumor cells, have been developed to provide minimally invasive approaches to monitor tumor evolution and detect recurrence. However, none of these new approaches are used in clinics due to their low specificity and/or sensitivity. Additionally, nomograms or predictive models have been created using biomarkers and clinicopathologic data to assess patients’ outcomes and survival. While significant progress has been made, the integration of melanoma biomarkers into routine clinical practice remains limited. This review summarizes current advancements in melanoma biomarkers, including traditional serum and immunohistochemical markers, as well as developments in molecular, genetic, circulating, and predictive biomarker approaches.

## Simple Summary

Clinical melanoma biomarker research is switching from the use of traditional blood and immunohistochemical markers to genetic and molecular biomarkers that could provide clinicians with improved prognosis and diagnosis. Despite the development of several traditional markers such as lactate dehydrogenase (LDH), melanoma inhibitory activity (MIA), and S100 calcium-binding protein (S100B), their low specificity and sensitivity limit their clinical use. Improvements in tumor genomics, like BRAF, KIT, and NRAS mutations, have enhanced risk stratification. Gene expression signatures have been created, such as SkylineDx (Merlin) and DecisionDx (Castle Biosciences), and are guiding surgical decisions and helping with risk of recurrence assessment. Despite the emergence of liquid biopsy markers, like microRNAs and circulating tumor DNA, none are clinically used due to sensitivity constraints and lack of validation. Nomograms using clinicopathological data combined with biomarkers have been developed to assess patients’ risk of recurrence and survival. This review summarizes these different approaches.

## 1. Introduction

Melanoma incidence rates have been rising globally and are a worldwide public health concern. Biomarkers have the potential to help with diagnosis, determine prognosis, guide treatments, and model surveillance schedules. There are different classifications of biomarkers, each with a unique insight into different aspects of each patient’s disease. Biomarkers can be non-invasive and can play a key role in helping clinicians with staging and classifying melanoma subtypes. Biomarkers are relatively novel in melanoma and might need more research to fully understand their potential. Our review focuses on exploring what is currently known about melanoma biomarkers and the importance of further study into what they can contribute clinically. To contextualize these advances, it is important to first consider the historical biomarkers that have traditionally guided melanoma diagnosis, staging, and monitoring. These established markers provide the foundation upon which newer molecular biomarkers are being developed.

## 2. Traditional Biomarkers in Melanoma

With the current clinical management of melanoma, traditional biomarkers are the predominant tools for clinicians to diagnose, stage, and monitor. These biomarkers are categorized into two groups: serum-based proteins, which includes lactate dehydrogenase (LDH), S100 calcium-binding protein (S100B), melanoma inhibitory activity (MIA), and tumor-associated antigen 90 immune complex (TA90-IC) [1,2] and immunohistochemical (IHC) markers of tissue such as S100 protein (S100), human melanoma black-45 (HMB-45), melanoma antigen recognized by T cells 1 (Melan-A/MART-1), and SRY-box transcription factor 10 (Sox10) [3,4,5] (Table 1).

### 2.1. Serum-Based Markers

#### 2.1.1. LDH

Lactate dehydrogenase (LDH) is a tetrameric cytoplasmic enzyme involved in the final step of glycolysis [12]. Melanoma cells are known to express the Warburg effect, where the cells are constantly undergoing high accelerated proliferation, driving a high glycolytic flux environment leading to an increased concentration of LDH [13,14]. As the tumor rapidly expands and outpaces the local blood supply, hypoxia triggers cellular necrosis; subsequently, the cell’s plasma membrane will burst, releasing LDH into the extracellular matrix and systemic circulation [15]. When quantifying the concentration of LDH, a standard serum assay is utilized, serving as a prognostic and management biomarker rather than a diagnostic tool [16]. As of 2026, LDH serves as the only serum-based marker that has been formally incorporated into the American Joint Committee on Cancer (AJCC) 8th edition staging system, specifically as a tool to classify stage IV patients [17]. It is well understood that measurements of LDH can assist in the management of therapeutic efficacy, tumor burden, and potential disease progression [18,19]. However, its poor diagnostic specificity restricts its clinical use. Elevated serum levels are commonly seen in non-cancerous conditions, such as tissue damage, myocardial infarction, acute hepatitis, and intravascular hemolysis, to name a few [15,20,21].

#### 2.1.2. MIA

Melanoma Inhibitory Activity (MIA) produced and secreted by melanoma cells in vitro is a small 11 kDa soluble protein that is not routinely utilized in U.S. clinical practice [22,23]. Research showed that MIA is a pro-metastatic factor [1]. It binds to extracellular matrix (ECM) components like laminin and fibronectin, covering the binding sites for integrin on the surface of melanoma cells, which allows tumor cells to detach from the ECM, resulting in invasion and systemic spread [24,25]. MIA can be measured in patients’ serum via enzyme-linked immunosorbent assay (ELISA) to assist in staging and monitoring therapy [24]. Several studies showed that increased levels of MIA could predict recurrence as early as 6 months earlier than clinical approaches, but have failed to translate into standard management protocols [1,24,26]. Despite MIA potentially being a valuable marker for stage III and IV disease, its low sensitivity in early-stage patients makes it unreliable; a study conducted showed it was only detectable in 13–23% of stage I and II patients [1]. Furthermore, increased MIA levels can be detected in non-malignant conditions, such as rheumatoid arthritis and other degenerative joint diseases [27]. Additionally, increased levels of MIA have been reported in patients who experienced physical trauma or underwent athletic exertion [28]. MIAs’ inability to reliably detect low tumor burdens in early-stage patients creates a gap in the detection window, allowing the disease to progress undetected [1].

#### 2.1.3. TA90-IC

Tumor-Associated Antigen 90 Immune Complex (TA90-IC) is a 90 kDa glycoprotein that, as of 2026, is not utilized routinely in the U.S. [2]. Reported in approximately 70–75% of melanomas, its antigen lacks malignancy specificity as it is present in other cancers, including lung, colon, and breast. Historically, TI90-IC is quantified in the serum via ELISA in an investigative cohort to evaluate for risk stratification and prognosis after wide excision in patients with stage I to III melanoma or as a predictor for subclinical metastatic disease and patient survival outcomes [2,29,30]. Early longitudinal studies demonstrate that serum-based TA90-IC ELISA can identify recurrence on average 19 months earlier than routine evaluations, such as x-rays and physical clinical examinations [29,30]. The TA90-IC assay offers a great predictive window into disease recurrence but failed to translate into a wider array of clinical implementation, as it shows variable sensitivity, which is highly dependent on the biophysical state of the antigen and the disease stage. This was commonly seen with patients in advanced stage IV disease; free circulating TA90 antigen would saturate available antibodies, disrupting the antigen–antibody ratio, rendering the results of the assay variable and clinically uninterpretable [29]. Early studies report that sensitivity and specificity for the detection of metastasis in stage I patients are approximately 77% and 76%, respectively [2]. Jones et al. recommended the use of TA90-IC in combination with other site-specific markers or clinical imaging to improve specificity and confidence in diagnosing, as TA90-IC cannot function as an independent diagnostic tool [2]. 

### 2.2. Immunohistochemical Markers

#### 2.2.1. S100B

S100 calcium-binding protein B (S100B) is a low-weight calcium-binding protein that functions as a dual-entity biomarker used in both circulating serum assays and as a tissue immunohistochemical (IHC) stain [31,32,33]. S100B regulates multiple intracellular processes, such as differentiation, cell cycle progression, and inhibition of tumor suppressor p53 [34,35]. Melanoma cells actively secrete S100B, serving as a highly sensitive indicator of melanocytic activity [32,33]. Known to promote tumor cell survival and malignant progression, S100B functions as a cytokine when extracellular levels become elevated [36]. It is well documented that S100B concentrations will rise before clinical and/or radiological evidence of disease relapse; international protocols, specifically across Europe, utilize longitudinal serum S100B tracking in advanced staging and monitoring [33,37,38]. Studies have shown that S100B showed increased sensitivity compared to LDH in detecting progression from stage III to stage IV. Várvölgyi et al. demonstrated that S100B had significantly higher sensitivity than LDH (61% vs. 31%) when detecting disease progression [19]. Despite its use in Europe, S100B possesses unfavorable qualities that have limited its adaptation into U.S. clinical practice. This is due to its lack of diagnostic sensitivity in low tumor burden environments, specifically in early stages (I and II), where it routinely fails to alter physiological baseline levels [37].

#### 2.2.2. HMB-45

Human Melanoma Black-45 (HMB-45) is a mouse monoclonal antibody that assists dermatopathologists as an immunohistochemical diagnostic tool [3]. Studies show that this antibody targets the gp100 (Pmel17) protein, a type 1 transmembrane glycoprotein [39]. Gp100 protein is an important biological component of pre-melanosomes, as it forms the fibrillar matrix [40,41]. Due to HMB-45 only reacting with the glycosylated form of gp100, it makes a highly specific marker [41]. With nearly 100% specificity, HMB-45 is regarded as the gold standard to confirm melanocytic differentiation. Unlike S100, HMB-45 does not stain neural-crest-derived cells, such as neurofibromas and Schwann cells, nor dendritic cells in lymph nodes. HMB-45 is currently used as a confirmatory staining marker for diagnosis [3,42]. While benign melanocytic nevi lose HMB-45 expression as they progress deeper into the dermis, expression of the lesion can assist pathologists in determining malignancy vs benign disease [42,43,44]. However, HMB-45 is not expressed in certain histological variants like spindle cells and desmoplastic melanoma [45]. Because of this, HMB-45 is often paired with markers like SOX10 and S100 for diagnostic purposes [3,5,46,47].

#### 2.2.3. Melan-A/MART-1

Melanoma Antigen Recognized by T cells (MART-1), otherwise known as Melan-A, is an important protein involved in trafficking and formation of melanosomes [48,49]. Identified as a target for cytotoxic T lymphocytes in melanoma patients, its primary utility has been used as a contemporary diagnostic immunohistochemical marker for primary melanoma diagnosis [4,50]. Studies showed a sensitivity rate of 80–90% or greater [42]. Like S100, it serves as a “pan-melanoma” marker but with increased specificity, not staining Schwann cells or neurofibromas [42,51]. Like HMB-45, Melan-A has limitations; its expression is constantly lost in melanoma subtypes like spindle cell and desmoplastic melanomas, leading to potential false negatives [45]. Additionally, it has been reported that Melan-A can stain capsular nevi within lymph nodes or “pseudo nodes,” which can be mistaken for micrometastatic deposits [52]. Hence, Melan-A is commonly incorporated into a multi-marker panel [53].

#### 2.2.4. Sox10

SOX10 is a nuclear regulator required for maturation, maintenance, and specification of neural crest-derived cells, melanocytes, and glial cells. Identified as a key member of the SRY-related HMG-box (SOX) family of transcription factors [54,55,56]. SOX10 is a nuclear marker that has a significant diagnostic advantage over S100, as it provides a clearer and more distinct immunohistochemical (IHC) signal and demonstrates a sensitivity rate of 97–100% in primary and metastatic lesions [5]. Hence, SOX10 has emerged as the premier “pan-melanoma” marker for various melanoma subtypes. SOX10 is commonly used to identify challenging histological variants, such as desmoplastic melanoma and spindle cells [5,57]. Due to its high specificity, SOX10’s most important application is to detect micrometastases in sentinel lymph nodes (SLNs) by highlighting isolated clusters of metastatic cells without cross-reacting with other surrounding nodal components [5,58]. However, studies have also documented the expression of SOX10 in non-melanocytic malignancies [59]. Due to its diagnostic shortcomings, guidelines dictate that its use be accompanied by other specific cytoplasmic markers [5,57].

Despite their clinical utility, traditional serum and immunohistochemical biomarkers have a wide range of sensitivity and specificity, particularly low in early-stage disease. These limitations (Table 1) have prompted the development of molecular and genetic biomarkers that provide deeper insight into tumor biology and enable more precise prognostic and therapeutic stratification.

## 3. Molecular and Genetic Biomarkers 

The development of newer biomarkers has evolved rapidly over the past few decades, progressing from simple serum markers to complex profiling that incorporates genomic and transcriptomic analyses. These advancements have the potential to identify melanoma patients, predict disease prognosis, and inform treatment decisions. In particular, molecular biomarkers such as gene signatures have shown the capability to differentiate melanoma from non-malignant tissue while concomitantly predicting responses to immune checkpoint blockade treatments [12]. While showing promising results, molecular and genetic biomarkers remain at the exploratory level due to a lack of validation, specificity, and/or sensitivity.

### 3.1. Tumor Markers

Several tumor markers provide insight into tumor biology, such as mutations in the mitogen-activated protein kinase (MAPK) signaling pathway and tumor suppressor alterations like neurofibromin-1 (NF1) and cyclin-dependent kinase inhibitor 2A (CDKN2A). These alterations contribute to melanoma progression and prognostic stratification.

#### 3.1.1. Genomic Alterations

Genomic alterations, such as B-Raf proto-oncogenes (BRAF), Neuroblastoma RAS viral oncogene homolog (NRAS), or KIT proto-oncogene receptor tyrosine kinase (KIT) mutations within the MAPK pathway, were among the earliest biomarkers used in clinical settings. The MAPK signaling pathway plays a central role in melanoma pathogenesis, and mutations can cause uncontrolled cell production, limit apoptosis, and contribute to treatment resistance.

BRAF is a serine/threonine kinase located downstream of RAS in the MAPK pathway. BRAF mutations such as V600E are common, occurring in approximately 40–50% of cutaneous melanoma patients, and have led to the development of targeted therapies called BRAF/MEK inhibitors such as vemurafenib, which has demonstrated improved response rates and overall survival (OS) outcomes in stage 4 melanoma patients [60]. These targeted therapies bind to the BRAF ATP-binding site and suppress the MAPK pathway, thereby decreasing tumor cell production. BRAF inhibitors are always used with MEK inhibitors to reduce the activation of alternative growth pathways. While BRAF/MEK inhibitors can have temporary benefits by reducing tumor cell proliferation, tumor cells often develop resistance to these therapies through reactivation of the MAPK signaling pathway or by using alternative signaling mechanisms [13]. Among currently identified genomic alterations in melanoma, BRAF mutations remain the most clinically actionable due to the availability of BRAF-targeted therapies approved by the FDA, such as vemurafenib (with cobimetinib), which focuses on BRAF mutations and others like dabrafenib with trametinib and encorafenib combined with binimetinib [14].

NRAS is a GTPase that functions upstream from BRAF and plays a major role in sending signals from cell surface receptors. NRAS mutations are less common than BRAF mutations, occurring in approximately 15–30% of melanoma patients, yet are frequently associated with a more aggressive disease phenotype and poorer clinical outcomes [15]. These mutations usually lead to persistent downstream signaling and uncontrolled cell proliferation. While BRAF mutations tend to only affect MAPK pathways, patients with NRAS mutations tend to have worse outcomes because these mutations can affect multiple pathways such as MAPK, phosphoinositide 3-kinase-AKT (PI3K-AKT), and the Ral guanine nucleotide dissociation stimulator (Ral-GDS) [16]. This broader signaling activation makes NRAS mutations particularly difficult to address, as there are multiple pathways that would need treatment. Thus, the development of targeted inhibitors for NRAS mutations has been more challenging than for mutations affecting BRAF pathways. As a result, there are limited therapeutic strategies focused on targeting NRAS mutations. Although NRAS mutations contribute significantly to a better understanding of melanoma biology, in comparison to BRAF mutations, effective targeted NRAS therapies remain limited, with MEK inhibitors having limited clinical benefits [18].

Another type of mutation affecting the MAPK signaling pathway involves KIT mutations, which occur less frequently than BRAF and NRAS mutations. KIT encodes receptor tyrosine kinase (RTK) CD117, which contributes to the activation of the MAPK downstream pathway. KIT mutations such as D816V tend to be found in approximately 1–5% of melanomas overall and are primarily in subtypes of melanoma such as acral and mucosal melanoma, suggesting an association with distinct clinical characteristics [19]. This suggests the importance of molecular profiling in patients with atypical melanoma variants. KIT mutant melanomas are a particularly unique biomarker because they are closely associated with specific melanoma cases, making therapies targeting KIT mutations less broadly applicable. However, of the patients who could benefit from targeted tyrosine kinase inhibitor therapy, those with mutations in specific exons have had significant clinical activity. In a phase II clinical trial, nilotinib—a KIT-specific inhibitor—has been found to help in disease control in patients with KIT mutations [20]. These findings reinforce the clinical relevance of developing KIT-specific treatments and emphasize the necessity of integrating molecular profiling into treatment decisions, especially for those with certain melanoma subtypes. Currently, KIT mutations are typically associated with subsets of melanoma, like in acral or mucosal melanoma cases, where tyrosine kinase inhibitors can have some clinical benefit in select patients, although these responses are variable and less generalizable than BRAF treatments [21].

Other genomic alterations have been described, such as NF1 and CDKN2A, but have not resulted in the development of specific therapies or IHC markers. NF1 encodes neurofibromin, a tumor suppressor that regulates RAS signaling. Therapies targeting NF1 mutations are particularly difficult to develop because these mutations result in hyperactivation of the MAPK pathway as a whole, not just alteration of RTKs or G-Protein Coupled Receptors (GPCRs) [17]. This has caused therapeutic efforts to be focused on downstream pathway inhibition rather than directly trying to fix NF1 mutations. Similarly, CDKN2A mutations also result in a loss of function rather than a targetable protein receptor. CDKN2A encodes a tumor suppressor protein, p16, that regulates the cell cycle and, when mutated, leads to excessive cellular division. Studies evaluating the role of CDKN2A mutations have demonstrated that the loss of tumor suppressor function indicates invasive behavior of later-stage melanoma, supporting its role as a marker of disease progression [22]. Germline mutations are different than somatic mutations seen in CDKN2A (p16) and NF1. Collectively, these findings illustrate that although mutations such as NF1 and CDKN2A can provide useful prognostic information, therapeutic efforts can only treat complications that arise due to these mutations rather than developing direct targeted treatments to fix the mutations. In comparison to BRAF mutations, which are directly actionable, NF1 and CDKN2A mutations function mainly as prognostic biomarkers. Their relevance lies predominantly in helping clinicians better understand therapeutic resistance and tumor aggressiveness. In contrast to BRAF mutations, which remain directly actionable due to the availability of FDA-approved targeted treatments, alterations such as NRAS, KIT, NF1, and CDKN2A function mainly as prognostic biomarkers with limited therapeutic targeting options.

#### 3.1.2. Gene Expression Signatures

Genomic biomarkers have helped with treatment decisions but are typically limited to specific clinical scenarios and are not equally applicable across all patient populations. Consequently, there has been increasing interest in gene expression biomarkers as they may offer broader clinical applicability and can provide valuable information in assessing tumor behavior. Analyses of RNA expression in melanoma patients can provide a comprehensive assessment of tumor progression and can be used to stratify patients with melanoma into different groups depending on their disease staging and metastatic risk [23]. For example, transcriptomic analyses have identified expression signatures capable of distinguishing primary from metastatic melanoma, including models such as the CAM_121 gene expression profile that stratifies patients according to metastatic risk [24]. These patients are usually separated into either class 1 or class 2, with class 1 being those with low-risk melanoma and class 2 being those with more aggressive forms of melanoma, typically leading to worse health outcomes. This stratification framework can be useful in aiding with risk assessment to help patients better understand their prognosis, as well as helping surgeons distinguish between high and low-risk patients. Other gene expression signatures, such as interferon gamma cytokines (IFN-γ), have been shown to predict response to programmed cell death protein 1 (PD-1) checkpoint blockade by identifying inflammatory responses in tumor microenvironments [25]. These findings highlight the ability of gene expression signatures to reflect tumor immune activity and enhance prognostic accuracy for better individualized care management.

Despite these advances, clinical integration of gene expression profiling remains limited. There are gene-pair signature biomarkers that can help to better understand one’s response mechanisms, such as programmed death ligand 1 (PDL-1). Higher levels of PDL-1 expression on tumor and immune cells have been associated with increased response rates to blockade therapies, yet its clinical use has been limited due to its low sensitivity and heterogeneous expression across tumors [26]. Although many gene expression signatures have demonstrated prognostic relevance, most remain investigational, with further validation required before full clinical implementation. These limitations highlight the need for more comprehensive approaches to better capture the complexity of melanoma tumors to better predict treatment responses. Although gene expression signatures are useful in developing risk stratification for patients, their usage has largely been limited to research efforts rather than clinical applications. According to the National Comprehensive Cancer Network (NCCN) guidelines, gene expression signatures have less clinical impact compared to clinicopathologic factors such as age, Breslow thickness, ulceration and primary tumor location [27]. This explains why gene expression signatures are currently limited in clinical utilization until further research can provide standardized validation.

Among currently available assays, the Castle Biosciences 31-gene expression profile (31-GEP) has demonstrated utility in stratifying cutaneous melanoma patients into low and high-risk metastases groups. Clinical studies have suggested associations between the 31-GEP classifications and sentinel lymph node risk positivity. These studies have focused on low-risk nodal metastasis, which can help physicians to make more educated decisions regarding the use of sentinel lymph node biopsies (SLNB) based on tumor aggressiveness and potentially reduce unnecessary procedures [28]. A recent panel supported the integration of the 31-GEP into clinical settings based on prior research, which has demonstrated its efficacy with prognostic melanoma decisions [29]. However, NCCN guidelines currently do not recommend routine use of 31-GEP testing to guide surgical decisions with sentinel lymph nodes. Recently, population-based SEER analyses have raised concerns about the reliability of considering the 31-GEP testing for SLNB decision. One study demonstrated that 82.5% of patients with +SLN were classified as low risk (Class 1A) by the 31-GEP assay [30]. These findings demonstrated that false reliance on the 31-GEP test can lead to inaccurate surgical decisions in patients who were predicted to be low risk yet later were found to have +SLN. This indicates that the use of 31-GEP should be used with caution as part of a multidisciplinary evaluation rather than a replacement for standard clinicopathologic assessment. In contrast, the Merlin clinicopathologic and gene expression profile (SkyLineDX) assay has achieved limited guideline-recognized use in select melanoma patients undergoing consideration for SLNB. While the Castle 31-GEP primarily serves as a prognostic tool for stratifying patients based on recurrence and metastasis risk, the Merlin assay was designed specifically to estimate SLN positivity by incorporating molecular and clinicopathologic variables. Current NCCN guidelines note that the Merlin assay may be considered an adjunctive tool in select patients with clinical stage IB melanoma (T1b-T2a) when a 10% SLNB positivity risk is considered acceptable during SLNB decisions [31]. Studies have shown that SLNB plays an important role [32]. Collectively, these findings demonstrate the evolving but still limited role of gene expression profiling in melanoma management and the importance of validation studies to determine which should be used for informing prognostic and surgical decisions (Table 2).

#### 3.1.3. Other Molecular Tumor Biomarkers

Chemokines actively reflect the tumor microenvironment as they regulate inflammation, immune cells, and angiogenesis. One chemokine in particular that has been studied in relation to melanoma is CXCL1. Elevated CXCL1 levels in tissue have been shown to contribute to immunosuppression by recruiting myeloid suppressor cells and neutrophils, a mechanism that has been shown to have a significant role in melanoma progression [34]. Accordingly, CXCL1 has emerged as a potential prognostic biomarker that can complement the molecular models currently used to better assess melanoma immune evasion. Unlike static genomic alterations, CXCL1 reflects an active role in the tumor environment throughout a patient’s journey. By using transcriptomics, CXCL1 expression can be evaluated by identifying key circulating components, supporting its potential as a minimally invasive biomarker. It has also been found that RNA expression can be associated with melanoma and distinguish patients into groups at either low or high risk for metastases [23]. These findings suggest that CXCL1 may reflect dynamic tumor immune interactions during the progression of melanoma. 

Recent advances in melanoma biomarkers have demonstrated that melanoma progression is dynamic and requires more than just static timepoint biomarkers. Increasing research has demonstrated that melanoma cells undergo phenotypic adaptation in response to therapeutic exposure and other factors. This is also referred to as phenotype switching, where melanoma cells transition between proliferative and invasive cellular states, causing increased therapeutic resistance and disease progression. As a result, biomarkers capable of capturing longitudinal tumor evolution could be more useful in comparison to static biomarkers based on tissue obtained at diagnosis. Adaptive stress response pathways have emerged as important regulators of melanoma aggressiveness and treatment resistance, with recent studies highlighting the role of calcium channel-dependent stress signaling in melanoma cells. Alterations in intracellular calcium signaling pathways have been associated with increased melanoma cell proliferation, metastatic potential, and resistance to targeted therapies [35,36]. Metabolic reprogramming has also been implicated in melanoma adaptation by allowing tumor cells to survive under hypoxic conditions, promoting resistance to therapeutic stress [33]. Recent metabolic profiling studies have also demonstrated that there are metabolism-related gene signatures seen in melanoma that also reinforce the role of metabolic adaptation in melanoma [37]. Another potential regulator in melanoma cells is the androgen receptor (AR) signaling, which has been implicated in tumor proliferation and melanoma aggression. Both non-genomic and genomic AR signaling pathways appear capable of influencing melanoma plasticity and adaptive stress response. Furthermore, recent evidence has shown that ligand activation of AR can increase melanoma invasiveness while increasing resistance to mediated cytotoxicity, suggesting that AR signaling influences both tumor aggression and immune evasion in melanoma [38]. Collectively, these findings support the use of longitudinal biomarkers in melanoma due to dynamic changes throughout disease progression for both therapeutic and immune pressures over time that static biomarkers may struggle to appropriately address.

The role of immune evasion biomarkers regarding innate immune system interactions has gained increased attention in melanoma, with ligands such as UL16 binding protein 1 (ULBP1) and natural killer group 2D (NKG2D) demonstrating predictive significance in melanoma. These ligands participate in tumor immune surveillance and natural killer (NK) cell recognition. It has been found that dysregulation and shedding of NKG2D ligands have been associated with impaired NK responses, thereby facilitating immune escape within the melanoma microenvironment [39]. Addressing these concerns, research has shown that therapeutic options aimed at increasing the antitumor activity of peripheral blood NK cells may help combat melanoma immune escape and improve responses to immunotherapy [40]. Emerging evidence further suggests that oxidative stress pathways and adaptive tumor stress responses may regulate expression of these ligands, linking increased immune evasion to tumor adaptation and microenvironment remodeling in melanoma. 

While genomic and transcriptomic biomarkers offer critical insight into tumor-intrinsic characteristics, they are often derived from tissue samples and may not fully capture the dynamic and systemic nature of melanoma progression. As a result, increasing attention has been directed toward circulating biomarkers, which can be detected in peripheral blood and provide a minimally invasive approach to monitoring tumor behavior in real time.

### 3.2. Blood-Derived and Other Markers

#### 3.2.1. Role of Circulating miRNAs in Melanoma Biology

MicroRNAs (miRNAs) are small non-coding RNA molecules, about 18–25 nucleotides long, that control gene expression after transcription by breaking down mRNA or blocking its translation. In melanoma, changes in miRNA levels are important for tumor growth, progression, immune escape, and spread. Unlike many markers found inside cells, miRNAs are very stable in the bloodstream, especially when they are inside extracellular vesicles like exosomes or bound to protein complexes, including high-density lipoproteins [41,42]. MicroRNAs can be found in many body fluids, such as serum [43,44], plasma [45], cerebrospinal fluid, urine, breast milk, and tears [46]. Early research showed that miRNAs stay stable in plasma even after long storage or repeated freeze–thaw cycles, and they can be measured reliably in blood samples. These features make them promising as minimally invasive cancer biomarkers.

Research has shown that melanoma cells release tumor-related miRNAs into the bloodstream, where they can indicate tumor activity. These circulating miRNAs can act as oncogenes, helping cells grow and invade, or as tumor suppressors, slowing tumor growth and spread. For instance, miR-21 and miR-155 help melanoma cells survive and affect immune responses, while miR-211 and miR-125b are linked to less tumor growth and spread [47,48]. MiRNAs also affect immune responses in the tumor environment by controlling how immune cells develop, how T-cells are activated, and how cytokines signal [49,50]. These effects can change how well immune checkpoint therapies work, since certain miRNAs influence tumor immune escape and response to treatment [51]. Because immunotherapy is so important in treating melanoma, circulating miRNAs are promising biomarkers that show both tumor changes and immune activity.

#### 3.2.2. miRNA Expression Signatures and Prognostic Value

MiRNA expression signatures are made of several miRNAs whose combined levels give useful clinical information. These signatures have been investigated as potential tools to help diagnose melanoma, assess risk, predict recurrence and survival, detect metastasis, and forecast how patients will respond to treatment.

Several studies have shown that miRNA-based signatures can tell melanoma patients apart from healthy people and predict how the disease will progress. For example, a panel of five miRNAs was found to predict outcomes in uveal melanoma, with changes in miRNA levels linked to the risk of metastasis and OS [52]. In cutaneous melanoma, panels of three to ten circulating miRNAs have shown similar diagnostic value, with better sensitivity and specificity than single serum biomarkers [48].

Research on miRNA signatures has also looked at predicting the risk of recurrence and how the disease progresses. Wiggins et al. found that profiles of tumor-derived miRNA expression were linked to clinical outcomes in stage II and III melanoma patients. This suggests that circulating miRNA signatures may eventually help identify people at higher risk of recurrence who might need closer monitoring or extra treatment [53]. In addition, inherited miRNA variants have been linked to how patients respond to immune checkpoint inhibitors and the chance of immune-related side effects. For example, certain miRNA changes are connected to responses to anti-CTLA-4 therapy and the risk of immune-related side effects [54]. These results suggest that testing for circulating miRNAs could help guide treatment choices and reduce unnecessary side effects in melanoma care.

It is still difficult to accurately assess the risk of recurrence in early-stage melanoma, which makes these findings important. Besides helping predict outcomes, inherited miRNA variants have shown value in predicting how patients will respond to immune checkpoint inhibitors and their risk of immune-related side effects. Weidhaas et al. found certain inherited miRNA changes linked to responses to anti-CTLA-4 therapy and immune-related side effects [54]. As a result, testing for circulating miRNAs could help guide treatment decisions and lower the risk of unnecessary side effects in melanoma patients.

Some studies have combined miRNA signatures with other molecular or clinical factors to improve prediction accuracy. Using miRNA expression data together with transcriptomic profiles or clinical information has led to better sensitivity and specificity than using miRNA signatures alone. This is likely because it gives a more complete picture of both tumor biology and immune responses [48]. However, most miRNA signatures have no or limited multicenter validation, high variability in testing methodologies, and a lack of reproducibility across diverse patient cohorts. Currently, there have not been any circulating miRNA signatures that have been able to achieve sufficient prospective validation or standardization to be implemented in routine melanoma care. Therefore, while miRNA signatures hold potential as minimally invasive biomarkers for precision oncology, further standardization and large-scale validation studies are required before clinical implementation.

#### 3.2.3. Circulating Tumor DNA

Circulating tumor DNA (ctDNA) consists of fragments of tumor DNA released into the blood when cells die. It can be used for early cancer detection, tracking treatment response, spotting recurrence, and finding resistance mutations [55,56]. ctDNA carries tumor-specific mutations and can help identify the genetic type of melanoma. Given that melanoma tumors often have mutations like BRAF and NRAS, ctDNA has begun to emerge as a promising noninvasive investigational approach for monitoring diseases in real time. Detectable ctDNA has been shown to strongly correlate with tumor burden and treatment response. Increasing ctDNA levels can appear months before radiographic progression, allowing earlier detection of recurrence [57]. In metastatic melanoma, ctDNA changes are linked to response to immune checkpoint inhibitors. Patients who clear ctDNA quickly during anti-PD-1 therapy have better progression-free survival, while persistent or rising ctDNA levels are linked to treatment resistance [58,59].

Despite these benefits, ctDNA is not currently considered part of the standard of care in clinical practice and remains investigational outside of select research and academic settings. The main challenges are low sensitivity in early-stage melanoma, variation in the amount of ctDNA released in the blood and lack of standard detection thresholds. Technical differences between testing platforms and the need for extremely sensitive detection methods also make it harder to use ctDNA widely in clinics [55,65].

#### 3.2.4. Circulating Tumor Cells

Circulating tumor cells (CTCs) are whole melanoma cells that break away from primary or metastatic tumors and enter the bloodstream. Their presence shows that cancer can spread through the blood and has the potential to metastasize.

In melanoma, higher numbers of circulating tumor cells (CTCs) are linked to more advanced disease and worse survival. Studies show that patients with detectable CTCs have much shorter progression-free and OS than those without CTCs [66,67]. Analyzing CTCs may provide important biological information about mutations and how the cancer changes, which matters in melanoma because of its variety and resistance to treatment. However, technical limitations, low detection sensitivity, and lack of assay standardization continue to challenge implementation into routine clinical care. While CTC analysis is not yet a routine part of melanoma care, it is a promising area for future research, especially for tracking cancer spread and understanding treatment resistance.

#### 3.2.5. Exosomes and Extracellular Vesicles

Exosomes are tiny vesicles released by melanoma cells that carry DNA, RNA (including miRNAs), proteins, and lipids, reflecting the tumor’s molecular features. These vesicles help the tumor grow by promoting immune suppression, new blood vessel formation, and the preparation of sites for metastasis. For example, exosomes expressing PD-L1 can suppress T-cell activation and are linked to how patients respond to immune checkpoint inhibitors. High or increased levels of exosomal PD-L1 are associated with poorer responses to anti-PD-1 therapy, while decreasing levels during treatment may reveal a benefit [42]. Due to the abundance in the blood and their capability to protect their contents, exosomes are promising biomarkers. Exosomal signaling may contribute to positive benefits, such as immune escape, tumor plasticity, and preparation of the pre-metastatic niche. However, the lack of standard methods for isolating and quantifying them limits their use in clinics.

#### 3.2.6. Tumor-Educated Platelets (TEPs)

Tumor-educated platelets are platelets whose RNA profiles have been altered through interaction with tumor cells. Melanoma cells can transfer RNA molecules into platelets, effectively reprogramming their transcriptome. TEPs have been proposed as a potential indicator of tumor presence, as platelets actively scavenge tumor-derived molecules in circulation. RNA sequencing of TEPs has demonstrated the ability to distinguish cancer patients from healthy individuals with high accuracy across multiple tumor types, including melanoma [68]. However, their role in melanoma management remains investigational and would require substantial validation.

#### 3.2.7. Strengths and Limitations

Blood-based biomarkers have several benefits for managing melanoma. They allow for minimally invasive monitoring of tumor biology and make it possible to take repeated samples over time, which helps track how the tumor changes and how it responds to treatment [55,69].

Additionally, circulating biomarkers can show the diversity of tumors, since they may come from several tumor sites instead of just one biopsy [56]. Also, biomarkers such as ctDNA and circulating miRNAs have shown promise for early detection of recurrence and tracking treatment response, especially during immunotherapy [48,58]. However, there are still several limits to using these biomarkers in clinics. Differences in how samples are collected, processed, and tested can make it hard to get consistent results across studies [65]. Sensitivity is also a problem in early-stage disease, since low tumor burden can mean biomarker levels are too low to detect [55]. Biological complexity also makes interpretation harder. Circulating miRNAs can originate from different cell types, such as immune or stromal cells, and ctDNA levels can be influenced by the amount of tumor DNA released and patient factors [41,42,56].

Overall, circulating biomarkers offer promising minimally invasive approaches for monitoring melanoma progression, treatment response, and tumor evolution. Among these, ctDNA currently has the strongest translational evidence, especially in advanced-stage disease and immunotherapy monitoring. However, most circulating biomarkers, including circulating miRNA signatures, circulating tumor cells, exosomes, and tumor-educated platelets, remain investigational due to a lack of assay standardization, limited prospective validation, and inconsistent reproducibility across studies. Because of this, these circulating biomarkers are not currently incorporated into routine standard melanoma care.

## 4. Nomograms and Predictive Models

### 4.1. Nomograms and Predictive Models

Nomograms are mathematical models used to demonstrate a relationship between two or more variables and can help predict patient outcomes for diseases such as cancer. Many nomograms include visual charts or graphics that facilitate computation of a mathematical function, making them easily accessible to both medical professionals and the general patient population [70]. The first medical nomogram was created by L. J. Henderson in 1928 to describe blood composition as a physical–chemical substance and to represent the relationship between the many variables in maintaining equilibrium in a complex biological system [70]. Nomograms have evolved further to calculate complex statistical variance to approximate patient disease states using computerized methods and visuals [71].

Nomograms have been developed to help physicians with melanoma diagnosis, staging, and treatment decisions. For early-stage patients, nomograms have been created to assess the necessity of a sentinel lymph node biopsy (SLNB) [72], to predict the sentinel lymph node (SLN) metastatic status [73] and to estimate the risk of recurrence or metastases [74,75,76] in advanced-stage melanoma patients. Nomograms have been developed to determine OS and to predict site-specific and age-specific recurrence [77,78,79].

### 4.2. Nomograms for Early-Stage Melanoma Patients

Nomograms have been designed to predict a person’s risk of developing melanoma. Vuong et al. [80] created a model using hair color, nevus density, first-degree family history of melanoma, previous non-melanoma skin cancer, and lifetime sunbed use as predictive factors. The model provides good discrimination with an Area Under the Curve (AUC) of 0.70 [95% CI, 0.67–0.73] using an internal cohort and of 0.63–0.67 on an external validation cohort. Another recurrence model, developed by Cust AE et al. [81], assesses the risk of second, third, and fourth primary melanoma and performs with a c-index of 0.73 [95% CI, 0.68–0.77], 0.65 [95% CI, 0.62–0.68], and 0.65 [95% CI, 0.61–0.69], respectively. The model itself uses a combination of demographic, phenotypical, histopathological, and genomic factors, and the amount of sun exposure to assess these recurrence risks in treated melanoma patients.

Historically, early-stage melanoma patients’ prognosis and risk of recurrence were determined using clinic-pathological features, especially SLN status. Despite a generally good prognosis, up to 20% of patients with tumor-negative SLN (-SLN) will develop metastases [82,83]. Therefore, several groups have created nomograms to assess the risk of recurrence and OS. For example, Chang et al. created a model to estimate the risk of recurrence in -SLN patients using age at diagnosis, sex, ulceration status, site of primary tumor, and Breslow thickness. Their model, based on 2668 patients, can assess the overall risk of recurrence as well as at 2-, 5-, 10- and 20-years after wide excision surgery. It performs with a c-index of 0.76 (95% CI: 0.74–0.79) overall and 0.87 (0.83–0.90) and 0.82 (0.78–0.85) at 1 and 2 years before remaining around 0.70 up until 20 years [84]. More recently, Janka et al. developed nomograms to predict the risk of recurrence at 3, 5, and 10 years at specific sites using Breslow thickness, ulceration status, primary tumor site, Clark invasion level, and other clinicopathological features. Their nomograms showed c-indexes above 0.80 for LN, lung, visceral and brain metastases in both their training and validation cohorts. Additionally, they showed differences in risk of recurrence amongst patients’ age ranges, with those over 70 having a lower risk of regional lymph node metastasis than those under 40 [79]. Some groups have created nomograms specific to certain subtypes of melanoma. For example, Shi et al. established a model for non-metastatic cutaneous melanoma of the limbs. Using 12 independent clinicopathological features, the model showed a C-index for OS of 0.853 (95% CI: 0.839–0.867) and of 0.922 (95% CI: 0.908–0.936) for cancer-specific survival [85].

Predicting the tumor status of the SLN remains one of the most important goals, as it can help with patients’ prognosis, treatment and SLNB procedure decision. Wong et al. developed the MSKCC (Memorial Sloan Kettering Cancer Center) model that determines the risk of having a tumor-positive SLN (+SLN). It is based on clinical features including patient age, site, and ulceration status of the primary tumor. The models had an AUC of 0.68 (*p* < 0.0001) [76]. The MSKCC model was then improved upon by the team of Lo et al. at the Melanoma Institute of Australia (MIA), creating the MIA nomogram. The MIA model removed the use of the Clark level and primary site present in the MSKCC model and added 3 new parameters: melanoma subtype, mitotic rate, and lympho-vascular infiltration (LVI). Using a cohort of 3477 patients to construct their model, compared to 979 in the MSKCC model, the MIA model showed a predictive accuracy of 73.9% [95% CI, 71.9–75.9%]. The MIA final model includes only 6 clinicopathological parameters and can predict the SLN status, potentially allowing for a reduction in the number of unnecessary SLNB [86]. Similarly, Bertolli et al. created a model predictive of a +SLN with the purpose of narrowing down the number of patients that require SLNB. This study showed that Breslow thickness, mitotic rate, histological regression, lymphatic invasion, site of primary lesion, and Clark level were all statistically relevant to predict SLN status. The nomogram reached an overall AUC of 0.751 [87]. More recently, Lourdault et al. [88] created a nomogram using clinicopathological features such as Breslow thickness, primary tumor site, histological subtype, and LVI. It performed with an AUC of 0.706 (0.661–0.751) on the MSLT-1 training cohort and of 0.715 (0.706–0.724) on the National Cancer Database (NCDB) validation data set. This is compared to an AUC of 0.723 (0.715–0.731) with the MIA model using the NCDB dataset.

### 4.3. Nomograms for Advanced-Stage Melanoma Patients

Many nomograms developed for advanced-stage melanoma patients are tailored to assess OS, risk of recurrence and predict responses to systemic therapy. Li et al. [89] created a nomogram evaluating the probability of cancer-specific death (CSD) for advanced-stage melanoma patients. Using 10 clinicopathological features such as age, sex, race, marital status, insurance, N stage, T stage, number of organs with metastases, surgical removal of metastases, and receipt of chemotherapy, they developed nomograms predictive of OS at 6, 12, and 18 months. The AUCs for these models ranged from 0.702 for 6-month OS to 0.700 for 18-month OS in the training cohort and from 0.702 for 6-month OS to 0.656 for 18-month OS. Similarly, Tropea et al. [90] created models for patients with +SLN who underwent complete lymph node dissection (CLND) to predict 3-, 5- and 10-year OS. The models were developed using clinicopathological factors of age, gender, primary melanoma site, Breslow thickness, ulceration status, sentinel node tumor burden, number of +SLN, and non-sentinel lymph nodes (NSN). They showed that the 3-year, 5-year, and 10-year OS rates were 79%, 70%, and 54%, respectively.

Responses to systemic treatment can also be predicted using nomograms, which could potentially determine the best treatments for each patient, especially for immunotherapies that have significant side effects. Pires da Silva et al. [91] created a nomogram to predict the response to various immunotherapy treatments in metastatic melanoma patients. Their nomogram includes the presence/absence of liver and lung metastases, serum LDH, blood neutrophil-lymphocyte ratio (NLR), the type of therapy (monotherapy/combination), and the line of treatment. They demonstrated an AUC of 0.68 for progression-free survival and an AUC of 0.77 for OS. This nomogram is an important tool to help physicians with treatment decision-making. Although immune checkpoint inhibitors (ICIs) have improved outcomes for some melanoma patients, they can also cause immune- related toxicity, making it important for physicians to know patients’ potential response and when these treatments are beneficial for the patient. The model developed by Lippenszky et al. [92] is currently the only one that specifically considers the potential side effects that can come from the use of ICIs. They created a predictive model capable of evaluating the risks of 3 types of immunotoxicity: pneumonitis, hepatitis, and colitis, as well as 1-year OS rates. Model AUCs for instances of toxicity across all cancer types were 0.739, 0.729, 0.755 and 0.752 for pneumonitis, hepatitis, colitis and 1-year OS, respectively [92]. A major concern for advanced-stage melanoma patients after receiving systemic treatment is recurrence, as it is generally associated with a very poor prognosis. Boutros et al. [93] created nomograms to assess relapse-free survival and distant metastasis-free survival (DMFS) in stage III melanoma patients undergoing immunotherapy treatment.

Models have also been developed to determine the risk of recurrence independently of the patient’s stage. Stassen et al. created models to predict both 5-year recurrence-free survival (RFS) and 5-year melanoma-specific survival (MSS) in melanoma patients of pT1b or higher classifications. This model was constructed using age, sex, presence of ulceration, primary tumor location, histological subtype, Breslow thickness, sentinel node status, number of sentinel nodes removed, maximum diameter of the largest sentinel node metastasis, and Dewar classification. With the validation cohort, the model performs with an AUC of 0.73 (0.71–0.75) for RFS and of 0.76 (0.74–0.78) for MSS [94].

### 4.4. Nomograms Combining Clinical Features and Molecular Markers

To improve the prognostic accuracy, sensitivity, and specificity of nomograms, several groups have combined clinicopathological factors with genetic or blood markers in their models. Zhang et al. [95] created a nomogram that combines 4 genes with 6 clinical factors and is able to predict 3- and 5-year OS in melanoma patients. This model performs with an AUC of 0.832 ± 0.071 with the validation cohort. Similarly, Tian and at., developed a nomogram predictive of OS using 3 long non-coding RNAs (lncRNA) and clinicopathological features. Using the 3 lncRNAs, they created a risk score that has AUCs for 3-, 5-, and 10-year OS of 0.638, 0.635, and 0.683 for the validation set, respectively. When they added the clinical features to the risk score, the nomogram performed with AUCs of 0.74, 0.621, and 0.621 in the validation set, for the 3-, 5-, and 10-year OS rate, respectively [96].

Gerami et al. [97] developed a 31-gene profile (31-GEP) discussed earlier in this manuscript that can accurately predict the risk of metastases in early-stage melanoma patients [23]. When the 31-GEP is combined with the standard AJCC staging system, it increases sensitivity for RFS, DMFS and OS. This shows that, when used in conjunction with the AJCC staging system, 31-GEP increases the accuracy of patient risk assessments.

Additionally, some nomograms for advanced-stage melanoma patients combined assess survival after immunotherapy treatment. For instance, Chatziioannou et al. [98] developed a model combining clinicopathological features and blood biomarkers such as LDH that can predict progression-free survival in unresectable stage IV patients receiving first-line anti-PD1 immunotherapy. However, the nomogram performs with a C-index of 0.67.

### 4.5. Clinical Significance and Accuracy of Nomograms

Many nomograms have been created, but only a few are currently used in clinics, such as the MIA nomogram that predicts SLN status and the need for SLNB in melanoma patients. However, its use remains controversial. Indeed, despite statistical effectiveness, a major concern is the potential lack of net benefits using nomograms. Hosein et al. [88,99,100] showed that the MSKCC nomogram only provides positive net benefits when the nomogram threshold is set at 5% and 9–10%. Similarly, the MIA nomogram only provides a net benefit with a 9% threshold and is potentially harmful to patients with thresholds of 5–8% and 10%. This study demonstrated that nomograms do not provide net benefits compared to all patients undergoing SLNB. A recent study by Drebin et al. [101] showed the limited clinical utility of both the MIA and MSKCC models. Both models demonstrated strong sensitivity rates (97.7% for MIA and 95.9% for MKSCC) but low specificity rates at clinically relevant thresholds (10.4% for MIA and 16.4 for MSK). The net reduction in avoidable SLNBs performed was assessed as –2.029 true negatives per 100 patients for MIA and –5.073 for MSK with risk thresholds of 5% and as 0.365 (MIA) and –3.734 (MSK) with a 10% risk threshold. These results showed that more patients underwent an unnecessary SLNB when using these nomograms with a threshold of 5–10% compared to treatment for all. Performing an SLNB on any patient with T1b melanoma or higher according to the AJCC Staging System provided consistently better results compared to both the MSK and MIA models at 5% and 10% risk thresholds, demonstrating the limited net clinical benefit of these nomogram models [101]. Similarly, Lourdault et al. [88] showed that only 3% of the patients are spared from SLNB when using a clinically relevant risk cut-off of 0.05 with their model, making the clinical application of nomograms questionable. This also highlights the lack of specificity of nomograms. Indeed, to avoid missing potential patients with +SLN, a low threshold is needed, which creates a high sensitivity while reducing the specificity, leading to low true negative rates and many false positives. Additionally, nomograms tend to be marginally effective in only specific sub-populations of patients instead of being effective for all melanoma patients. Indeed, the MSKCC model is more accurate in predicting +SLN in older patients, while the MIA model is more effective for patients with high mitotic rate [100].

Currently, the adoption of nomograms in clinics remains limited, as suggested by Mayhew et al. [102]. Despite this, the future of nomograms in the clinic promises earlier diagnosis and prognosis for melanoma patients both at early and advanced stages. The ability to detect and track the survival and progression of disease in melanoma patients could improve treatment decision-making and potentially patients’ outcomes. Nomograms that combine gene expression profiles and/or miRNA signatures with clinicopathological features could potentially improve nomograms’ specificity and sensitivity, pushing them beyond the accuracy of current tools.

## 5. Clinical Integration

Despite some limitations such as low specificity, LDH is the only traditional biomarker currently used in clinics [103]; LDH is part of the AJCC staging system. The other traditional biomarkers, such as MIA, S100B, or Sox10, are not used due to several limitations as described in Table 1.

Using circulating biomarkers in melanoma care could greatly improve patient outcomes by allowing earlier detection of recurrence, better tracking of treatment response, predicting who will benefit from immunotherapy, and personalizing follow-up plans. Of these biomarkers, ctDNA currently has the most evidence for use in clinics. Changes in ctDNA levels during immunotherapy have been linked to treatment response and progression-free survival in patients with metastatic melanoma [58,59]. Circulating miRNAs may also help guide clinical decisions by showing both tumor activity and immune responses. Since miRNAs control immune signaling, they are being studied as predictors of response to immune checkpoint inhibitors [48,51]. In the future, combining ctDNA, miRNA signatures, and clinical features may give better predictions than any single biomarker. However, before these can be used routinely, large validation studies and standard testing methods are needed to make sure results are reliable [65,104] (Table 3).

Although circulating biomarkers provide valuable insights into tumor dynamics and treatment response, their clinical utility depends on how effectively this information can be integrated into decision-making frameworks. To address this need, predictive tools such as nomograms have been developed to combine biomarker data with clinical variables and generate individualized risk assessments.

## 6. Conclusions

Biomarkers in melanoma have transitioned from traditional serum and immunohistochemical markers to molecular, circulating, and imaging-based biomarkers that provide unique insight into the progression of each patient’s diagnosis. While markers like HMB-45 and S100B have played a key role in melanoma diagnosis, their limited specificity has highlighted the need for more precise biomarker development. These developments have been seen in key areas such as molecular and genetic biomarkers, with Merlin and Castle both having assays that have already been implemented clinically. These assays have helped with stratifying patients and predicting tumor responses to better understand the disease behavior of a patient. New forms of biomarkers such as circulating miRNAs and tumor DNA are emerging because of their ability to capture tumor dynamics in real time. Circulating biomarkers have already demonstrated many benefits, like early detection of recurrence and helping to make more informed immunotherapy decisions. Predictive nomograms are imagining biomarkers that are used in conjunction with clinicopathological factors to better understand individualized risk assessment. Overall, biomarkers have made substantial progress in their involvement in melanoma care, especially on the clinical side. However, future directions should focus on the standardization of these biomarkers and on using multiple biomarkers to help clinicians make better-informed decisions for each individual patient. Such integration has the potential to enhance diagnostic accuracy, optimize treatment selection, and ultimately advance the practice of care in melanoma.

## Figures and Tables

**Table 1:** Reasons for the limited clinical involvement of traditional markers.

Marker	Reason for Limited Clinical Involvement
LDH [6]	Low specificity to melanoma Elevated levels in non-malignant patients
MIA [7]	• Limited clinical utility that can be associated with other biological processes ○ Infection. ○ Inflammation. ○ Other cancers.
TA90-IC [8]	• Limited prognostic value due to false positives corresponding to inflammatory states ○ Hepatitis.
S100B [9]	Low specificity for early-stage patients Poor metastases detection rates in stage III patients compared to F-FDG-PET/CT imaging.
HMB-45 [10]	Low specificity for malignant melanoma in SNL compared to Melan-A Poor sensitivity in desmoplastic melanoma detection is important.
Melan-A/MART-1 HMB-45 [10,11]	Not suitable for screening. No expression in advanced/metastatic lesions. Unreliable for longitudinal tracking.
Sox10 [11]	Unable to differentiate cutaneous melanoma from benign melanocytic nevi.

**Table 2:** Comparison of Merlin and Castle gene expression assays in Melanoma.

Feature	Castle Biosciences (DecisionDX) [61,62,63]	Merlin Assay (SkyLineDX) [32,64]
Test type	Gene expression signature and clinicopathological features	Gene expression signature and clinicopathological features
Composition	31 genes + clinical factors	8 genes + Breslow thickness + patient’s age
Primary goal	SLN positivity prediction and risk of recurrence	SLN positivity prediction
Target patients	AJCC Stage I-III	Early-stage (T1-T2)
Clinical Integration	2015	2020 Only the NCCN-recognized test for SLNB risk
Risk stratification	Class 1 (low) vs. Class 2 (high)	Low vs. high SLN risk
Clinical setting	Early-stage melanoma	SLNB evaluation
Key benefit	Identifies low-risk patients	Reduces unnecessary SLNB
Use case	Limited clinicopathologic data available	SLN decision-making
Outcome impact	Improved risk stratification	Improved surgical selection

**Table 3:** Summary of established, clinically used adjunctive and investigational biomarkers.

Biomarker Type	Name	Relevance
**Established Biomarkers**		
Serum Marker	LDH	Serum marker incorporated into AJCC staging assists with prognosis, disease progression and tumor burden.
Genomic Alterations	BRAF mutation	Guides the use of BRAF/MEK targeted therapy
Gene Expression Signatures	Merlin assay (SkyLineDx)	NCCN guideline-recognized use. Assist in decision-making regarding SLNB.
**Adjunct Biomarkers**		
Serum Marker	S100B, MIA, TA90-IC	Used for prognosis and monitoring of disease. Not routinely used in U.S. clinical practice.
IHC Markers	S100, HMB-45, Melan-A, SOX10	Used as diagnostic adjuncts for melanoma, histopathological characterization of tissue samples.
Genomic Alterations	NRAS, NF1, CDKN2A	Prognostic assessment, understanding of tumor biology.
Prognostic nomograms	MIA nomogram	Incorporates molecular and clinical variables for risk and survival prediction. Limited real-world adoption
Gene Expression Signatures	Castle Biosciences (DecisionDx)	Not recognized as an SLNB decision-making substitute
**Investigational Biomarkers**		
Molecular Tumor Biomarkers	CXCL1, Chemokines	A potential prognostic biomarker lacks validation.
Circulating biomarkers	miRNA signatures ctDNA, CTCs, Exosomes, TEPs	Promising non-invasive biomarkers but lacking reproducibility, validation and having low sensitivity in early-stage disease. Could potentially be used for diagnosis, prognosis, survival and response to treatment. Not yet clinically actionable
Biomarkers associated with innate immune escape and various pathways	Calcium channel-dependent stress signaling, Androgen receptor signaling, stress-associated ligands such as ULBP1 and NKG2D	Capable of capturing longitudinal tumor evolution to follow disease progression, assess aggressiveness and immune modulation. Have potential prognostic and predictive significance in melanoma.

## Data Availability

No new data were created or analyzed in this study.

## Abbreviations

LDH	lactate dehydrogenase
S100B	S100 calcium-binding protein
MIA	melanoma inhibitory activity
TA90-IC	tumor-associated antigen 90 immune complex
IHC	immunohistochemistry
HMB-45	human melanoma black-45
Melan-A/MART-1	melanoma antigen recognized by T cells 1
Sox10	SRY-box transcription factor 10
MAPK	mitogen-activated protein kinase
NF1L	neurofibromin-1 (NF1)
CDKN2A	cyclin-dependent kinase inhibitor 2A
BRAF	B-Raf proto-oncogenes
NRAS	Neuroblastoma RAS viral oncogene homolog
KIT	KIT proto-oncogene receptor tyrosine kinase
RTK	receptor tyrosine kinase
GPCRs	G-Protein Coupled Receptors
IFN-γ	interferon gamma cytokines
PD-1	programmed cell death protein 1
PDL-1	programmed death ligand 1
NCCN	National Comprehensive Cancer Network
31-GEP	31-gene expression profile
SLNB	sentinel lymph node biopsies
miRNA	MicroRNAs
CTLA-4	Cytotoxic T-lymphocyte-associated protein 4
ctDNA	Circulating tumor DNA
CTCs	Circulating tumor cells
TEPs	Tumor-educated platelets
AUC	Area Under the Curve
SLN	sentinel lymph node
-SLN	tumor-negative SLN
+SLN	tumor-positive SLN
MSKCC	Memorial Sloan Kettering Cancer Center
MIA model	Melanoma Institute of Australia model
LVI	lympho-vascular infiltration
CSD	cancer-specific death
CLND	complete lymph node dissection
OS	Overall Survival
NCDB	National Cancer Database
ICIs	immune checkpoint inhibitors
RFS	recurrence-free survival
MSS	melanoma-specific survival
lncRNA	long non-coding RNAs
AR	Androgen Receptor
NK cells	Natural Killer cells
NKG2D	Natural Killer Group 2D
